# Scale‐up economics for cultured meat

**DOI:** 10.1002/bit.27848

**Published:** 2021-06-17

**Authors:** David Humbird

**Affiliations:** ^1^ DWH Process Consulting Centennial Colorado USA

**Keywords:** animal cell culture, bioreactor design, fermentation, techno‐economic analysis

## Abstract

This analysis examines the potential of “cultured meat” products made from edible animal cell culture to measurably displace the global consumption of conventional meat. Recognizing that the scalability of such products must in turn depend on the scale and process intensity of animal cell production, this study draws on technoeconomic analysis perspectives in industrial fermentation and upstream biopharmaceuticals to assess the extent to which animal cell culture could be scaled like a fermentation process. Low growth rate, metabolic inefficiency, catabolite inhibition, and shear‐induced cell damage will all limit practical bioreactor volume and attainable cell density. Equipment and facilities with adequate microbial contamination safeguards have high capital costs. The projected costs of suitably pure amino acids and protein growth factors are also high. The replacement of amino‐acid media with plant protein hydrolysates is discussed and requires further study. Capital‐ and operating‐cost analyses of conceptual cell‐mass production facilities indicate economics that would likely preclude the affordability of their products as food. The analysis concludes that metabolic efficiency enhancements and the development of low‐cost media from plant hydrolysates are both necessary but insufficient conditions for displacement of conventional meat by cultured meat.

## Graphical abstract

This analysis examines the potential of “cultured meat” products made from edible animal cell culture to measurably displace the global consumption of conventional meat. The bioreactor design, process design, and production cost implications of scaling an animal cell‐culture process like a fermentation process are investigated.

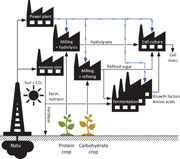

## 1 INTRODUCTION

**Figure 1:**
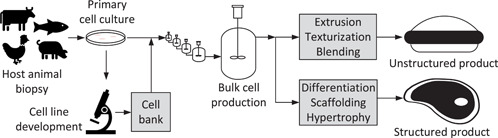
Conceptual cultured meat production process

“Cultured meat” refers to a nascent field of bioproducts that aim to replace conventional meat produced by farming and slaughter with analogous or alternative products made from edible animal cell culture. In one concept (Figure 1), cells from a live‐animal biopsy are propagated through a series of increasingly large bioreactors, growing in number with each step and ultimately inoculating a 20 m^3^ bioreactor to produce a batch of 2–3 tons of animal cell slurry (van der Weele & Tramper, 2014). The cultured cell mass, perhaps blended with vegetable proteins and fats, is further processed into unstructured mincemeat‐ or nugget‐style foods. Advanced concepts propose to deposit cultured animal cells onto an edible scaffold that provides form and possibly hypertrophy, resulting in structured food products that more closely resemble a cut of meat. Alternatively known as “cell‐based” or “cultivated” meat, these technologies are positioned to address global problems associated with industrial animal farming, such as its contributions to pollution, foodborne illness, and anthropogenic climate change (Chestney & Nebehay, 2019; Gerber et al., 2013; World Wildlife Fund, 2017).

From Figure 1 it can be concluded that the scalability of either class of products depends on that of the bulk cell production step. As further indicated in the figure, there is an expectation that this step would be carried out in large stainless‐steel tanks, as in a large fermentation plant. Precedent for this concept comes from the biopharmaceutical industry, where therapeutic proteins are produced in suspension cultures of recombinant mammalian cell lines in stainless‐steel bioreactors up to ∼20 m^3^. Scaling animal cell culture like a fermentation process, however, presents several technical and economic challenges. Animal cells proliferate much more slowly than microbial cells. Metabolically unregulated cells in vitro tend to exhibit inefficiencies that cause them to produce growth‐inhibiting catabolites such as lactate and ammonia. Mass‐transfer limitations are expected in large bioreactors, where gas sparging and agitation are limited by the potential for shear‐induced damage to animal cells, which lack a rigid cell wall. The capital costs of equipment and facilities with sterility safeguards adequate to avoid microbial contamination are likely to be high. Formulations of amino acids and protein micronutrients (growth factors) suitable for cell‐culture media are not currently produced at scales consistent with food production and are also understood to be rather expensive.

These technical and economic aspects are explored in a recent assessment of cultured meat's potential to measurably displace the human consumption of conventional meat (Humbird, 2020). Its methods and findings are summarized in the present article. Basic industrial fermentation and bioreactor design rules are used to establish a stoichiometry of mammalian cell growth and attainable cell density as a function of bioreactor size. Cost projections for media components and sterile bioprocessing equipment are developed. These insights are used to develop production cost estimates for conceptual fed‐batch and perfusion facilities that produce bulk animal cell mass. Suspension culture and conventional stainless‐steel construction are assumed for process design purposes. To set a global demand for media component costs, these facilities are considered within a larger market of 100 kTA (kilotonne per annum) of wet animal cell mass—similar to ascendant plant‐based meat replacements.

## 2 TECHNO‐ECONOMIC METHODS

### 2.1 Model cell and growth characteristics

Different animal cell types have been proposed for cultured meat production: embryonic or pluripotent stem cells, adult or mesenchymal stem cells, and primary cells (Ben‐Arye & Levenberg, 2019). Each of these would have characteristic proliferation and/or differentiation phases, each with its own media composition and bioreactor design. These details are presently unknown in the public sphere. Much more is known about mammalian cell lines used in biopharmaceutical manufacturing, most of which derive from Chinese Hamster ovary (CHO) cells. This analysis therefore draws on the CHO literature for guidance on cellular metabolism, growth inhibition, bioreactor design, and other aspects.

This analysis considers an abstract mammalian cell line adapted for suspension culture. Cells are assumed to be spherical with 70% intracellular water and a hydrated mass of 3000 pg. Cell growth proceeds with a maximum growth rate *µ*_max_ = 0.029/h, equivalent to a doubling time of 24 h. A CHON formula for animal dry cell mass (DCM_a_) of CH_1.68_O_0.34_N_0.21_ derives from an average composition of 15% lipid, 10% carbohydrate, 5% RNA/DNA, and 70% protein (Alberts, 2002). Sulfur, phosphorous, and metals are ignored and the CHON formula for protein (CH_1.57_O_0.31_N_0.28_) derives from an average amino acid profile of 207 cellular proteins (Xie & Zhou, 2005). Formation energies of ∆*H*_f_ = −73.0 kJ/mol and ∆*G*_f_ = −31.9 kJ/mol are estimated with correlations (Battley, 1999; Burnham, 2010).

An anabolic growth reaction for DCM_a_ is constructed from the macromolecular composition: Lipid and carbohydrate are synthesized from glucose (Glc); nucleotides are synthesized from glucose and one of the *N* atoms on glutamine (Gln), rejecting glutamate (Glu); and protein is synthesized from its individual amino acid residues (Xie & Wang, 1994). Guan and Kemp (1999) characterized catabolic stoichiometry for CHO cultures by matching catabolite formation rates with observed heat dissipation. Reaction 1 was deduced at the end of the batch, after cell growth had ceased:

(1) $$\begin{array}{lll} 1.0\text{Glc} & + & 0.13\text{Gln}+1.275O_{2}\to 0.26{\text{NH}}_{3}+1.77\text{Lac}+1.34{\text{CO}}_{2}\\ & & +\;0.95H_{2}O. \end{array}$$

This reaction is a superposition of respiration, glycolysis, and glutamine catabolism, and it can be generalized with two degrees of freedom, for example, lactate/glucose ratio (Lac/Glc) and glutamine/glucose ratio (Gln/Glc). As written, these ratios are relatively high (Lac/Glc = 1.77 and Gln/Glc = 0.13), indicating significant metabolic inefficiency consistent with the so‐called Warburg effect, which is frequently observed in rapidly proliferating animal cells (Hosios et al., 2016). Catabolism proceeds at a rate that meets an observed heat dissipation or “metabolic power.” West et al. (2002) showed that in vitro metabolic power *P*_M_ (in pW) can be related to hydrated cell mass *M*_c_ (in pg) with $P_{M}=0.148M_{c}^{0.75}$. The metabolic power of 3000 pg cells is 60 pW/cell and Reaction 1 (∆*H*_r_ = −681 kJ/mol) proceeds at 0.0077 mol/mol DCM_a_‐h. With *µ* = 0.029/h, anabolism and catabolism can be combined into an overall reaction:

(2) $$\begin{array}{l} 0.333\text{Glc}+0.342O_{2}+0.007\text{Arg}\;+\;0.004\text{Cys}+0.055\text{Gln}\\ \;+0.003\text{His}+0.007\text{Ile}+0.010\mathrm{Lys}+\;0.002\text{Met}\\ \;+0.005\text{Phe}+0.009\text{Thr}+0.002\text{Trp}+0.005\text{Tyr}\\ \;+0.010\text{Val}+0.013\text{Ala}+0.006\text{Asn}+0.008\text{Asp}\\ \;+0.011\text{Gly}+0.011\text{Leu}+0.007\text{Pro}+0.010\text{Ser}\\ \;\to 1{\text{DCM}}_{a}+0.005\text{Glu}+0.070{\text{NH}}_{3}+0.474\text{Lac}\\ \;+0.435{\text{CO}}_{2}+0.495H_{2}O. \end{array}$$

Given the metabolic inefficiency inherent to Reaction 2, it will stand in for an unoptimized “wild‐type” cell line. It will be demonstrated below, however, that its Lac and NH_3_ generation rates preclude it from reaching an economically high cell density. Growth optimization strategies include: extensive characterization; selection for lactate reuptake; transfection of glutamine synthetase enzyme; and feedback control of glucose and pH (Freund & Croughan, 2018; Pereira et al., 2018). To permit a 20 m^3^ fed batch carried out with this metabolism to remain under likely inhibition limits of Lac and NH_3_ (to be discussed shortly), the maximum inefficiencies are rather Lac/Glc = 0.50 and Gln/Glc = 0.025. Reaction 3 therefore represents this “metabolically enhanced” cell line:

(3) $$\begin{array}{l} 0.147\text{Glc}+0.378O_{2}+0.007\text{Arg}\;+\;0.004\text{Cys}+0.022\text{Gln}\\ \;+0.003\text{His}+0.007\text{Ile}+0.010\text{Lys}\\ \;+\;0.002\text{Met}+0.005\text{Phe}+0.009\text{Thr}+0.002\text{Trp}\\ \;+0.005\text{Tyr}+0.010\text{Val}+0.013\text{Ala}+0.006\text{Asn}\\ \;+0.008\text{Asp}+0.011\text{Gly}+0.011\text{Leu}+0.007\text{Pro}\\ \;+0.010\text{Ser}\to 1{\text{DCM}}_{a}+0.005\text{Glu}+0.004{\text{NH}}_{3}\\ \;+0.041\text{Lac}+0.455{\text{CO}}_{2}+0.613H_{2}O. \end{array}$$

**Figure 2:**
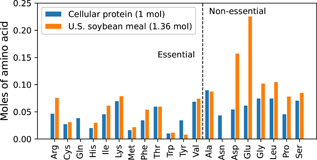
Amino‐acid profiles of cellular protein and U.S. soybean meal [Color figure can be viewed at wileyonlinelibrary.com]

At food scale, plant protein hydrolysates may be more cost‐effective and sustainable than amino acids produced individually by fermentation. Figure 2 compares the amino‐acid profile implied in Reaction 1 to that of U.S. soybean meal (U.S. Soybean Export Council, 2015). The essential amino acid (EAA) profiles are similar enough that if a quantitative hydrolysate of soybean meal were fed at 1.36 mol per mol protein (to match on threonine), all EAA requirements could be met except for glutamine and about 75% of tyrosine. With aggregate compounds standing for the soy hydrolysate and the unused amino acid fraction (UAA), the following enhanced‐metabolism reaction can be derived:

(4) $$\begin{array}{l} 0.147\text{Glc}+0.378O_{2}+0.022\text{Gln}+0.004\text{Tyr}\\ \;+0.192\text{Soy}\text{Hydr}.(C_{4.81}H_{9.49}O_{2.68}N_{1.28})\\ \;\to {\text{DCM}}_{a}+0.004{\text{NH}}_{3}+0.041\text{Lac}+0.455{\mathrm{CO}}_{2}\\ \;+0.613H_{2}O+0.142\mathrm{UAA}(C_{2.63}H_{4.86}O_{1.75}N_{0.60}). \end{array}$$

### 2.2 Cell density limitations

**Figure 3:**
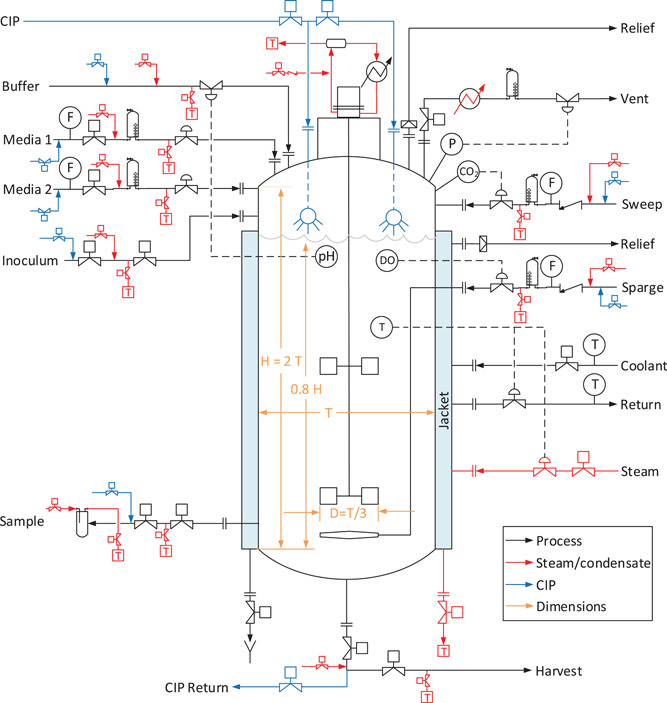
Schematic of a STR with external cooling jacket. Bioreactor diameter is denoted T and impeller diameter is D. Aseptic piping and instrumentation follow BPE‐2016 (ASME, 2016). STR, stirred‐tank bioreactor [Color figure can be viewed at wileyonlinelibrary.com]

Figure 3 presents a sketch of a stirred‐tank bioreactor (STR) with two impellers, jacket heating/cooling, and a final working volume of 80%. Sparged gas bubbles are used to transfer O_2_ into solution and strip CO_2_ out. The stirring action of the impeller enhances this gas‐liquid mass transfer. Sparging is quantified as superficial velocity *u*_s_ (m/s) and agitation as power input per unit of liquid volume (*P*/*V*, in W/m^3^). The maximum cell density supported in a STR may be limited by culture viscosity, gas‐liquid mass transfer rates, mixing time, catabolite accumulation rates, and other factors. As these limits are exceeded, the growth rate will drop precipitously due to inhibition. At the already‐low growth rate considered here, any inhibition is economically unacceptable with respect to the accumulation of bulk cell mass. In general, it will be more cost‐effective to stop the batch and begin a new one at the uninhibited growth rate.

#### 2.2.1 Viscosity

A practical maximum cell density in suspension culture occurs at a volume fraction *ф*∼0.25; above this limit, viscosity increases sharply as cell‐cell collisions become more frequent (Iordan, 2008). For 3,000 pg cells with a diameter of 17.7 μm, the absolute maximum attainable cell density is thus 86 × 10^6^/ml or 258 g/L wet. Note that for smaller cells like CHO, the maximum number density can be significantly higher (Clincke et al., 2013), but the maximum mass density is the same.

#### 2.2.2 O_2_ mass transfer

The volumetric oxygen transfer rate (OTR) is the product of a mass transfer coefficient *k*_L_ *a* and a driving force: the deviation of the dissolved oxygen concentration [O_2_] from its Henry's Law saturated concentration [O_2_*], expressed as a log‐mean difference over the bioreactor top and bottom:

(5) $$\text{OTR}=k_{L}a{\left([O_{2}^{*}]\;-\;[O_{2}]\right)}_{\text{lm}}.$$

To determine *k*_L_ *a*, the correlation of Xing et al. (2009) was developed for CHO cell‐culture media at 37°C:

(6) $$k_{L}a[s^{-1}]=0.075{\left(P/V\right)}^{0.47}{\left(u_{s}\right)}^{0.8}.$$

STR design rules for animal cell culture limit the gas sparge rate to *u*_s_ = 0.006 m/s, which is equivalent to 0.1 vvm in a 20 m^3^ bioreactor (Ozturk, 1996). Agitation is limited to a power input that creates turbulent eddies *λ*_K_ on the length scale of a single cell (∼20 µm):

(7) $$\lambda_{K}={\left(\frac{\nu^{3}}{50\left(P/V\rho \right)}\right)}^{1/4}.$$

where *ν* =* η*/*ρ* is the kinematic viscosity of the medium fluid and the factor of 50 corrects for the power input close to the impeller relative to the bulk *P*/*V* (Nienow, 2010). With sparging and agitation both at their recommended limits, a maximum attainable OTR can be estimated from Equations (5), (6), (7). Cell density is thus limited to the point where the culture's oxygen uptake rate is equal to this maximum OTR. To increase OTR (and thus cell density), oxygen or O_2_‐enriched air is typically sparged instead of air. This analysis assumes on‐site production of 90% O_2_ in a vacuum pressure‐swing adsorption unit.

#### 2.2.3 CO_2_ mass transfer

CO_2_ removal from the liquid phase follows a relation much like Equation 7. If CO_2_ does not accumulate in the liquid, then the CO_2_ transfer rate is approximately equal to the OTR. The liquid concentration of CO_2_ (typically measured as pCO_2_, or the CO_2_ partial pressure in equilibrium with the liquid phase) is therefore a function of the bioreactor sparge rate. In CHO culture, inhibition is noted when pCO_2_ falls outside a range of 40–100 mbar (Gray et al., 1996). With sparging fixed at *u*_s_ = 0.6 cm/s, the cell density must be limited such that the CO_2_ evolution rate does not cause pCO_2_ > 100 mbar.

#### 2.2.4 Mixing

Mixing time in the STR can be estimated with Equation 8 (Nienow, 2014). Here, *T* is the bioreactor diameter, *D* is the impeller diameter, and *H*_L_ is the liquid height. Mixing time should be less than 1/*k*_L_ *a* to ensure that dissolved O_2_ is quickly transported away from the bubble (Van't Riet & Van der Lans, 2011).

(8) $$\tau_{m}\approx 6T^{2/3}{\left(P/V\rho \right)}^{-1/3}{\left(D/T\right)}^{-1/3}{\left(H_{L}/T\right)}^{2.5}.$$

#### 2.2.5 Catabolite inhibition

**Figure 4:**
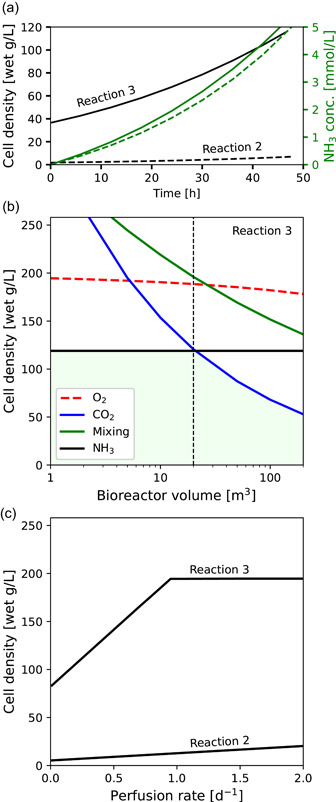
(a) Fed‐batch simulations in an O_2_‐sparged 20 m^3^ bioreactor with 80% max working volume and 5 mmol/L max NH_3_ concentration. Dashed lines: Reaction 2; solid lines: Reaction 3. (b) Maximum cell density achievable in fed‐batch suspension culture with Reaction 3. The limiting density for each constraint was computed independently of the others, and the density axis is truncated at the viscosity limit. (c) Maximum cell density achievable in perfusion suspension culture as a function of perfusion rate with Reactions 2 and 3 [Color figure can be viewed at wileyonlinelibrary.com]

In fed‐batch cell culture for biopharmaceuticals production, the accumulation of toxic and growth‐inhibiting catabolites is a far more frequently encountered limit than the physical limits discussed so far. Inhibiting concentrations of 2–10 mM NH_3_ have been reported for mammalian cells; lactate inhibition is an order of magnitude higher (Xie & Zhou, 2005). For modeling purposes, the limits of 5 mM NH_3_ and 50 mM lactate are considered here. Figure 4a (dashed lines) presents a fed‐batch simulation using Reaction 2 in a 20 m^3^ bioreactor sparged with 90% O_2_. After two cell mass doublings, the batch ends with 5 mM NH_3_ and a cell density of only 7.0 g/L. In fact, there is no practical way to reach an economically high cell density with a metabolism as inefficient as Reaction 2. At any appreciable starting density, NH_3_ inhibition would occur in a matter of hours. In a fed‐batch simulation with Reaction 3 (solid lines), a final cell density of 110 g/L is reached before NH_3_ inhibition.

These simulations can be extended to find the maximum fed‐batch cell density as constrained by O_2_, CO_2_, NH_3_, and mixing. As shown in Figure 4b, NH_3_ inhibition limits the cell density to 110 g/L in bioreactors <20 m^3^. At 20 m^3^, the NH_3_‐limited density is coincident with the pCO_2_‐limited density (the catabolic parameters of Reaction 3 were selected to cause this coincidence). Bioreactors >20 m^3^ have a lower maximum cell density due to CO_2_ inhibition. While NH_3_ inhibition can be mitigated to a degree with enhanced metabolic efficiency, CO_2_ inhibition cannot. Regardless of the metabolic parameters, the respiratory quotient (CO_2_/O_2_) of the growth reaction will remain ∼1 and the CO_2_ stripping rate will remain equal to (or less than) the O_2_ transfer rate. The only way to circumvent CO_2_ inhibition in a sparged bioreactor is to sparge harder, possibly to the point of cell death (Al‐Rubeai et al., 1995). This may preclude the scale‐up of animal cell culture into extremely large bioreactors.

CO_2_ and NH_3_ inhibition can also be mitigated, to a degree, with perfusion. In perfusion culture, the contents of the bioreactor are continuously cycled through a cell retention device, which continuously removes extracellular products and inhibitors and generally permits higher cell densities than fed‐batch cultures. At steady state, cells are bled from the bioreactor to maintain growth rate; in principle, this cell bleed could be a harvest stream for bulk cell culture. Perfusion culture volumes are limited by the capacity of the cell retention device. The alternating tangential‐flow (ATF) filter is commonly used in high cell‐density applications and the largest available (e.g., the Xcell ATF 10) can run at perfusion rates up to 1000 L/d (Pollock et al., 2013). In dual‐ATF configurations, 1 m^3^ bioreactors can thus be perfused at up to 2 reactor volumes per day (RV/d), and 2 m^3^ bioreactors at up to 1 RV/d. Figure 4c presents curves of attainable cell density as a function of perfusion rate. The wild‐type Reaction 2 generates 2 mmol NH_3_/mol DCM_a_‐h. If NH_3_ is removed via the perfusate stream at a steady‐state concentration of 5 mmol/L, then at a perfusion rate of 2.0/d it can be computed that inhibition is reached at a cell density of only 20 g/L (6.8 × 10^6^/ml). With the more efficient Reaction 3, an O_2_‐limited cell density of 195 g/L (65 × 10^6^/ml) can be achieved at a perfusion rate of 1.0/d.

### 2.3 Capital costs

The capital cost of a conceptual bulk animal cell‐culture process is developed from the bare‐equipment costs of its most important items. From this purchased equipment cost, a total capital investment (TCI) is obtained through the application of cost escalation factors, which are understood to be rather high for biopharmaceutical cell‐culture processes (Petrides, 2015). Compared to existing biopharmaceutical processes, future bulk cell‐culture processes for cultured meat would probably have similar safety and sterility requirements to protect the culture from being overcome by contaminating microorganisms or infected by adventitious viruses (Moody et al., 2011). Equipment and facility design, however, would necessarily be more commoditized. Process‐industry estimation software can thus be leveraged for capital cost development.

Figure 3 presents a sketch of a sterile STR suitable for animal cell culture: sterilizable inputs, jacket heating and cooling, CIP/SIP, automation, and so forth. The ASME standard for bioprocessing equipment (ASME, 2016) dictates full‐vacuum design and 316 L stainless‐steel construction. At 20 m^3^, Aspen Capital Cost Estimator (ACCE) estimates the cost of the bare vessel and agitator as ∼$330k, as indicated in Table 1. Piping and instrumentation costs can also be estimated with ACCE. With additions for surface treatment (electropolishing, passivation), internals (sparger, spray balls), and externals (exhaust heater, sterile impeller seal), the estimated total direct cost (TDC) of a 20 m^3^ system is $1.5 M. Estimated bare equipment (vessel and agitator) and total system costs for bioreactors of 1–200 m^3^ are further shown in Table 1, along with direct‐cost factors. While installation costs dominate at all volumes, a strong economy of scale is noted.

**Table 1:** Detailed costs for the sterile configuration in Figure 3 at 1 and 20 m^3^

1 m^3^			
ACCE vessel + agitator			$59k
ACCE piping			$201k
ACCE instr. + elec.			$454k
ACCE other direct cost			$3k
Add for internals/externals			$29k
Add for surface treatment			$28k
Total			$774k
20 m^3^			
ACCE vessel + agitator			$330k
ACCE piping			$360k
ACCE instr. + elec.			$476k
ACCE other direct cost			$22k
Add for internals/externals			$164k
Add for surface treatment			$132k
Total			$1.5M
*All volumes*			
	Bare	Installed	DCF^a^
1 m^3^	$59k	$774k	12.1
2 m^3^	$93k	$856k	8.2
5 m^3^	$138k	$966k	6.0
10 m^3^	$217k	$1.2M	4.4
20 m^3^	$330k	$1.5M	3.5
50 m^3^	$722k	$2.6M	2.6
100 m^3^	$1.3M	$4.0M	2.2
200 m^3^	$2.4M	$6.8M	1.8

*Note*: For volumes 1–200 m^3^, the bare equipment and total direct costs are shown.

^a^ Direct cost factor (Installed/Bare‐1)

To develop capital costs for specific configurations, TDCs for bioreactors are estimated with Equation 9. This piecewise correlation combines the ACCE estimates in Table 1 with estimates from SuperPro Designer at small volume, where ACCE is less accurate:

(9) $$\text{Cost}(\$k)=\left\{\begin{array}{cc} 30.7\times V+800 & V\geq 0.33m^{3}\\ 2285\times V+49.5 & V<0.33m^{3} \end{array}\right.\;.$$

Costs for minor process equipment items (media tanks, sterilizers, filters, etc.) are estimated with ACCE, SuperPro Designer, or the correlations in Couper et al. (2012). Process support equipment (utilities, O_2_ generation, etc.) is estimated independently or represented with an operating cost. To all equipment except bioreactors, an installation factor of 1.3× is applied to their purchased cost (Petrides, 2015). Building costs are computed from an estimate of equipment footprint and areal costs taken from ACCE or Petrides. To the TDC of the facility, an indirect cost factor of 0.6 is applied for engineering and construction fees, giving a total plant cost (TPC). An additional contingency factor of 0.15 is applied to the TPC to compute the TCI. Finally, the TCI is represented as an annual charge ($/y) by applying a capital charge factor (CCF; see Equation 10) of 15%/y to the TCI. Here, *i* is taken as 7.5% and *n* as 10 years; these are common values for food manufacturing facilities including plant‐based meat replacements (Damodaran, 2020; Maroulis & Saravacos, 2003).

(10) $$\text{CCF}[\%\;\mathrm{of}\;\mathrm{TCI}/y]=\frac{i}{\left(1-{\left(1+i\right)}^{-n}\right)}.$$

### 2.4 Raw material costs

Animal cell‐culture media generally contains a defined composition of sugar (glucose), up to 20 essential and non‐EAAs, fatty acids, phosphate, trace minerals, and various vitamins, hormones, and cytokines (collectively known as growth factors). Many of these components are not currently produced at scales consistent with food production. This section discusses where these raw materials might come from and how demand levels are likely to influence future price.

#### 2.4.1 Glucose

As the primary carbon and energy source in Reaction 5, glucose is required at 0.36 kg/kg of wet cell mass. Commercial d‐glucose (dextrose) is produced in the U.S. at corn wet mills and sold as corn syrup with a market volume of >4000 kTA. Contract prices for established glucose consumers are ∼$0.26/kg (USDA ERS, 2020). At this price, glucose is not anticipated to be a bottleneck to scale‐up, nor a significant contributor to production cost: only $0.24/kg wet cell mass.

#### 2.4.2 Amino acids

**Figure 5:**
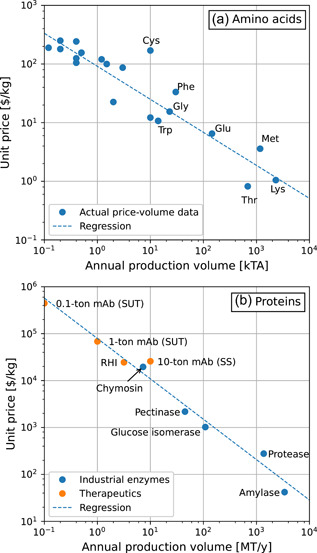
(a) Unit cost versus production rate for individual amino acids and their cost contribution to wet cell mass at scale (stoichiometry of Reaction 3). (b) Price‐volume relations for industrial enzymes and therapeutics. Adjusted to 2018$. RHI, recombinant human insulin; SS, stainless steel; SUT, single‐use technology [Color figure can be viewed at wileyonlinelibrary.com]

Each amino acid is currently produced at some commercial scale ranging from thousands of kTA for animal‐feed supplements to <1 kTA for aminos with primarily pharmaceutical uses. Figure 5a presents available price‐volume data for individual amino acids (BCC Research, 2017; IHS Chemical, 2019; Sanchez et al., 2018). Although Figure 5a clearly indicates that aminos with smaller market volumes cost more, the high‐volume/low‐cost data points for the major amino acids reflect feed‐grade formulations unsuitable for cell‐culture media. Suitably pure formulations cost more. Recognizing that the price‐volume relationship observed across all amino acids must also exist across formulations of an individual amino acid, the market volume of a novel “cultured meat‐grade” formulation is probably a more reliable predictor of price at scale than any specific details of its manufacturing process. These will certainly influence price, but not by multiple orders of magnitude. Prices for individual amino acids at scale are therefore correlated to estimated production volume by the following equation:

(11) log(Price[$/kg])=−0.563log(Prod.volume[MT/y])+3.65.

#### 2.4.3 Plant protein hydrolysate

Soybean hydrolysate was discussed above as a potential alternative source of amino acids. In animal‐free media formulations for cell culture, plant hydrolysates are sometimes used supplementally in otherwise chemically defined media (Babcock et al., 2007). Single amino acids are not generally produced from hydrolysates because posthydrolysis isolation is cost‐prohibitive. Instead, whole hydrolysates would have to be designed to provide all amino acids in the proper ratios (with the probable exception of glutamine, which decomposes easily). Current soybean meal pricing for animal feed is about $0.33/kg (USDA‐IL, 2020). Subtilisin protease enzyme at $15/kg (see Figure 5b) and 2% loading would further add $0.30/kg of meal processed. If meal were 48% protein, protein 88% soluble, and hydrolysis conversion 80%, then a formulation of mixed amino acids from hydrolysis could cost as little as $1.60/kg, plus processing costs. This analysis considers a price of $2/kg mixed amino acids.

#### 2.4.4 Protein micronutrients

Protein growth factors are provided in media to regulate growth and metabolism. Animal‐free sources of these proteins include highly processed extracts of plant proteins and recombinant proteins made by fermentation. Commercial recombinant proteins (therapeutics, industrial enzymes) have a price‐volume relationship similar to amino acids, as presented in Figure 5b and regressed in Equation 12 (Arbige, 1989; Gotham et al., 2018; Kelley, 2009).

(12) log(Price[$/kg])=−0.861log(Prod.volume[MT/y])+4.90.

Micronutrient usages are estimated on a loss basis: in perfusion culture, losses occur over time; in fed‐batch culture, losses occur when the bioreactor is emptied. Four distinct micronutrients are considered in the analysis: insulin at 19.4 mg/L, transferrin at 10.7 mg/L, fibroblast growth factor at 0.1 mg/L, and transforming growth factor β (TGF‐β) at 0.002 mg/L (Chen et al., 2011). Characteristic losses are, for example, 10–40 MT/y of insulin and 1–4 kg/y of TGF‐β. Although the unit prices estimated with Equation 12 are relatively high, growth factors only contribute $3–4/kg of wet cell mass at 100 kTA.

### 2.5 Fixed costs

Facility overhead is scaled to CAPEX and is here taken to comprise 4% TCI/y for maintenance (including CIP) and 5% TCI/y for insurance. Labor is quantified in terms of operator attention per batch, based on a series of task/time assumptions. A salary of $50,000/y for regular FTEs is taken from the U.S. Bureau of Labor Statistics—Chemical Plant Operator; the supervisor's salary is 140% of this. A labor burden of 100% is added to the total labor cost.

## 3 ANALYSIS RESULTS

### 3.1 Fed‐batch case study

**Figure 6:**
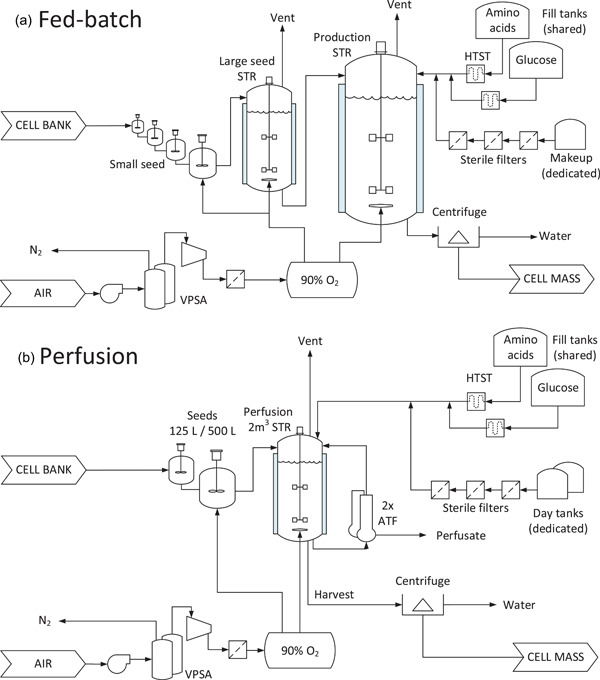
Process flow diagrams of conceptual bulk cell‐culture processes. (a) Fed‐batch. (b) Perfusion [Color figure can be viewed at wileyonlinelibrary.com]

A process flow diagram (PFD) of a conceptual fed‐batch cell‐culture process is given in Figure 6a. For the detailed example given below, the model cell‐culture facility is designed with 24 × 20 m^3^ production bioreactors and produces 6.8 kTA of wet cell mass. Cells are propagated from the lab through a seed train to the production bioreactors. Upon harvest, the cell mass is dewatered to 20% solids in a disk‐stack centrifuge. Two large media tanks attached to HTST sterilizers are shared between bioreactors to provide pre‐inoculation fill. A smaller, dedicated tank attached to sterile retention filters contains the pre‐mixed media that will be added during the batch.

The fed‐batch simulation in Figure 4a is used to size equipment and compute media and utilities usages. Bioreactor costs are estimated with Equation 9, while costs for the remaining equipment and buildings are estimated as described above. A Class 8 clean room is selected for cell‐culture areas and Class 6 for laboratory areas. The sum of equipment and buildings gives a TDC of $94 M. Indirect costs are factored from the TDC to give a TCI of $328 M. As an annual capital charge, this TCI is equivalent to $48 M/y, or ∼$12/kg of wet cell mass. At a total production volume of 100 kTA, macronutrients (amino acids) contribute an additional $19/kg and micronutrients (growth factors) $3/kg. Consumables (filter membranes), utilities (clean room power) and labor (95 total FTE) contribute a combined $3/kg. These capital and operating costs are summarized in Table 2. The overall cost of production estimated for a fed‐batch cell‐culture process is $37/kg wet cell mass.

**Table 2:** TEA estimates for the production of bulk cell mass by fed batch or perfusion

	Fed‐batch	Perfusion
Production rate (kTA)	6.8	6.9
Total bioreactor volume (m^3^)	649	197
Total FTE	95	132
*Capital costs*		
Production bioreactors	$34M	$83M
Seed bioreactors	$23M	$9M
Perfusion equipment	‐	$89M
Media prep	$17M	$41M
Dewatering	$4M	$2M
O_2_ PSA	$21M	$19M
CIP	$10M	$9M
Other equip	$22M	$43M
Production clean room	$40M	$49M
Lab clean room	$4M	$3M
Other buildings	$5M	$13M
Total direct cost	$178M	$360M
Engineering and construction	$107M	$216M
Fees and contingencies	$43M	$86M
Total capital investment	$328M	$663M
*Production cost contributors ($/kg)*		
Macronutrients	$19	$18
Micronutrients	$3	$3
Consumables	$1	$5
Utilities	$1	$1
Labor	$1	$2
Bioreactor CAPEX	$4	$6
Perfusion CAPEX	‐	$6
Buildings CAPEX	$3	$4
Rest of plant CAPEX	$5	$7
Total cost of production	$37	$51

**Figure 7:**
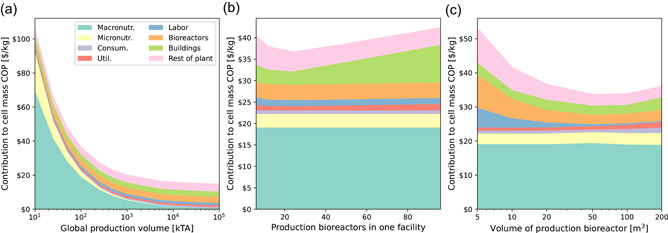
COP sensitivities predicted by the fed‐batch TEA model. (a) 24 × 20 m^3^ bioreactors at increasing global production volume. (b) Varying number of 20 m^3^ bioreactors in a single facility, at 100 kTA. (c) Varying volume of the production bioreactor (24×). COP, cost of production [Color figure can be viewed at wileyonlinelibrary.com]

Figure 7 presents sensitivity analyses for the fed‐batch production process. The chart is colored by the individual contributions of CAPEX, OPEX, and so forth, and the upper edge of the colored area represents the total estimated COP. In Figure 7a, nutrient costs vanish at extremely large production volume. An asymptotic cost of ∼$16/kg is predicted at 10^5^ kTA. At 100 kTA, substituting hydrolysate at $2/kg and repeating the fed‐batch simulation with Reaction 6 reduces the macronutrient contribution by almost $16/kg, bringing the total cost to $22/kg. Further opportunities for cost reduction are limited. Figure 7b indicates that 24 production bioreactors are optimal in a single facility; the production cost increases at >24 bioreactors because the clean room area grows faster than the process volume it contains. Figure 7c presents the sensitivity associated with the production bioreactor size, indicating an optimal volume of 50 m^3^. At larger volume, the reduction in final cell density (due to pCO_2_ limitations) outweighs the cost benefits of larger reactors.

### 3.2 Perfusion case study

A PFD of a conceptual cell‐culture process based on perfusion technology is given in Figure 6b. The 2 m^3^ production bioreactors are inoculated by expanding cells from the lab through 125 and 500 L seed bioreactors in fed‐batch mode. Upon inoculation, the production bioreactor also operates in fed‐batch mode up to 120 g/L. At this density, perfusion begins at a rate of 1.0/d (requiring 2× ATF 10 filters) and the retained cell density rises to 195 g/L per Figure 4c. Each production bioreactor operates with continuous cell harvest except during 10 scheduled turnarounds (72 h each) per year. The average time at steady state is ∼91%, and 9 kg/h of wet cell mass is harvested from each bioreactor. To produce 6.9 kTA (to match the fed‐batch process above), 96 bioreactors are required.

Equipment and building costs are estimated as described previously. Bulk amino‐acid and glucose tanks are shared for initial filling of all bioreactors, and each production bioreactor gets two dedicated tanks to hold 24 h of makeup media each. A cost for the ATF 10 perfusion devices is taken from Pollock et al. (2013). The TCI developed in Table 2 is $663 M, giving an annual capital charge of $97 M/y or ∼$23/kg wet cell mass. Macro‐ and micronutrient costs are similar to the fed‐batch process, while consumables costs are significantly higher due to the replacement of ATF membranes (20 per bioreactor per year at $16k each). The perfusion process is also slightly more labor‐intensive than the fed‐batch process (132 total FTE) and its overall cost of production is estimated as $51/kg wet cell mass.

**Figure 8:**
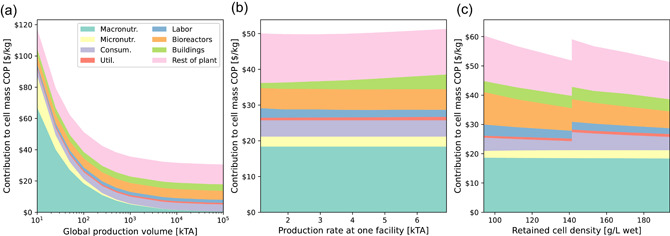
COP sensitivities predicted by the perfusion TEA model. (a) 6.9 kTA production in a single facility at increasing global production volume. (b) Varying production rate (i.e., number of bioreactors) at a single facility, at 100 kTA. (c) Increasing perfusion rate and cell density. At >1.0/d (140 g/L), a second ATF filter is added. ATF, alternating tangential‐flow; COP, cost of production [Color figure can be viewed at wileyonlinelibrary.com]

Figure 8 presents sensitivity analyses for the perfusion process. The limited volume, relatively high bioreactor direct costs, and the CAPEX and consumables associated with the perfusion device present significant disadvantages. Nutrient costs can be minimized by assuming a much larger global production volume, as shown in Figure 8a, or reduced $16/kg by substituting low‐cost hydrolysate. Figure 8b presents sensitivity to the facility production rate, noting a very weak minimum at 3.5 kTA. The issue with divergent clean room cost is also present for perfusion, but to a much smaller degree. In a 2 m^3^ bioreactor, a retained cell density of 195 g/L is attained at a perfusion rate of 1.0/d, which requires dual ATFs. With a single ATF and a perfusion rate of 0.5/d, 140 g/L of cells can be retained. As shown in Figure 8c, the capital and consumables costs associated with the second ATF offset the economic benefit of higher density, such that the production cost at 195 g/L is hardly any better than at 140 g/L.

## 4 CONCLUDING DISCUSSION

To reach a market of 100 kTA, or ten million consumers consuming 10 kg/y, it must be assumed that cultured meat has at least attained the price‐acceptance status of a reasonably affordable “sometimes” food. To assert a threshold on the subjective metric of affordability, this analysis submits a target of ~$25/kg of wet animal cell matter produced in a bulk growth step. After further processing, packaging, distribution, and profit, unstructured products made 100% from bulk cell mass at $25/kg might be expected to reach a minimum of $50/kg at the supermarket: The price of a premium cut of meat, paid instead for a mincemeat or nugget‐style product. Above this cost, the displacement of conventional meat by cell culture may arguably be measurable but increasingly less significant.

Although both estimates detailed above exceed this threshold, a fed‐batch process could potentially be brought under $25/kg with low‐cost hydrolysate media. The same is not true of the perfusion process, which has capital costs and capital‐dependent fixed costs that are well above the target. Hydrolysates appropriate for whole, unsupplemented cell‐culture media do not exist today and the assertions of their ultimate suitability and price are somewhat speculative. Further recall that both processes were examined with a cellular metabolism significantly enhanced relative to a wild‐type cell line, implying extensive characterization, process development, and metabolic engineering. From the modeling above, it can be concluded that metabolic efficiency and low‐cost hydrolysate media development can both be taken as necessary but insufficient conditions of affordability. Capital cost reduction is a secondary condition at best.

## AUTHOR CONTRIBUTION

David Humbird is the sole contributor to the article and the techno‐economic calculations described within.

## Supporting information

Supporting information.
